# Reply to: neuroprotection and haemodynamic stability: considerations for tailored anaesthesia in carotid endarterectomy: Considerations for tailored anaesthesia in CEA

**DOI:** 10.1097/EJA.0000000000002153

**Published:** 2025-05-07

**Authors:** Christian Vetter, Kathleen Seidel, David Bervini, Vladimir Krejci

**Affiliations:** From the Department of Anaesthesiology and Pain Medicine (CV, VK), the Department of Neurosurgery (SK, BD), Inselspital, University Hospital, University of Bern, 3010 Bern, Switzerland

Editor,

We thank Chiumiento and colleagues^1^ for their comments on our publication^2^ and agree with them that patients undergoing carotid endarterectomy (CEA) are elderly with a high-risk cardiovascular profile and often additional comorbidities. In our study, all patients were classified as ASA 3 or 4. Most of them had a symptomatic stenosis of the internal carotid artery with acute neurologic deficit(s), which in turn underlines the importance of anaesthesia management in conditions of critical cerebral blood flow.^2^

The debate between general anaesthesia versus regional anaesthesia has been addressed previously. We are aware that there are also nuances of both methods, which can be advantageous for selected cases. However, to date, no single anaesthesia method has been found to be superior over another, provided that cerebral perfusion is maintained and monitored. The ‘selected cooperative patient’ described by Chiumiento and colleagues for their general anaesthesia with intra-operative awaking (GAIA) represents an interesting example that allows clinical evaluation with a secured airway.^1^

Nevertheless, the goal of our study was not to evaluate different methods, but rather to refine an existing method in terms of improved haemodynamic and pharmacologic stability. Over the past years, our surgical, neurophysiology and anaesthesia teams have established a seamless collaboration during neurosurgical procedures, resulting in a very low rate of permanent deficits, even in high-risk patients. Continuous intra-operative neurophysiological monitoring (IONM) is a reproducible and validated method, which is not limited by neurological impairment, performance or cooperation. An additional point is continuous neurophysiological monitoring during the period of reperfusion in these patients, which is not given with GAIA. When neurophysiological monitoring of sensory-evoked potentials and motor-evoked potentials is performed during the entire procedure, including the postreperfusion period, defined warning criteria can be applied to allow outcome prediction. Thus, we benefit from quantifiable warning criteria to decide when to place a shunt and when not to.^3,4^

By adding dexmedetomidine, we have modified only one parameter. Reproducible IONM requires stable plasma and effect-site concentrations of hypnotics, including dexmedetomidine. Although we did not measure actual concentrations, PK-PD simulations predicted that our protocol would result in stable plasma concentrations of dexmedetomidine from the first measurement until reperfusion.

Although compelling human data in favour of propofol-induced burst suppression are lacking, this ‘nonevidence’ has largely been concluded from studies where ‘cerebral ischaemia’ was not a well controlled variable.^5^ On the contrary, a dose-dependent decrease of infarct size has been demonstrated after occlusion of the middle cerebral artery under propofol-induced burst suppression in rats.^6^ We are aware that direct translation of results from animal studies to humans must be evaluated with care, however, for obvious reasons, some animal studies cannot be reproduced in humans just for the sake of evidence. Thus, these experimental data are likely ‘the best’ that is currently available, and should not be dismissed as ‘obsolete’.

A protocol using TIVA with burst suppression, and adaptive management of cerebral perfusion using vasoactive drugs during cross-clamping has been routinely used in our institution. The published outcome data, which include morbidity and mortality, compare favourably to the existing literature. Intra-operative burst suppression and delirium has recently been found to *predict* postoperative delirium, but evidence for *avoidance* of intra-operative burst suppression using intra-operative EEG to decrease the risk of delirium is currently lacking.^7^ As reported in our manuscript, transient delirium occurred in less than 5% of our study population, which is significantly less than what has been found for other types of surgery.

To summarise, many approaches contribute to optimal patient care during CEA, highlighting the need for continued research in this area.
